# Are Alcoholism Treatments Effective? The Project MATCH Data: Response

**DOI:** 10.1186/1471-2458-5-76

**Published:** 2005-07-18

**Authors:** William R Miller

**Affiliations:** 1 Department of Psychology MSC03 2220, University of New Mexico, Albuquerque, New Mexico, USA 87131-0001; 2 Center on Alcoholism, Substance Abuse, and Addictions (CASAA), University of New Mexico, 2650 Yale Blvd. SE, Albuquerque, New Mexico, USA 87106

## Abstract

A response to Cutler RB, Fishbain, DA: Are alcoholism treatments effective? The Project MATCH data *BMC Public Health *2005, **5:75.**

## Text

Oh what a tangled web they wove! Cutler and Fishbain [1] re-analyzed Project MATCH data to challenge "a fundamental belief of addiction treatment that therapy is effective." They noted that patients who dropped out before receiving treatment nevertheless showed substantial change on the primary outcome measures, whereas only those who completed therapy showed significant further improvement during the 12-week treatment period. Across conditions, about two-thirds the change that would occur was already present at the beginning of treatment. From this, they concluded "that current psychosocial treatments for alcoholism are not particularly effective."

This phenomenon of early gain is indeed interesting, and by no means unique to the MATCH multisite trial. In alcohol treatment studies with weekly drinking measures, it is common to see substantial change appear very early in treatment, change that is sustained well across months or years of follow-up [2]. A majority of the reduction in drinking that will occur by the end of treatment is already present in the first few weeks of treatment, often in the very first week. Cutler and Fishbain interpreted this common finding as proving that treatment did not work, thereby explaining why "the results of the MATCH clinical trial were disappointing."

## Disappointing Findings?

Disappointment with study findings, of course, depends upon what the beholder had hoped to see in the first place. Overall improvement in MATCH patients was substantial and maintained quite well across three years of follow-up [3]. Main effects of treatments were never expected in MATCH; that is, no overall differences in efficacy had been predicted [4]. The surprise, then, was not the absence of between-treatment differences on the two primary outcome variables, but that on at least one other time-honored outcome measure – the percentage of patients maintaining complete abstinence – those in the Twelve-Step Facilitation treatment fared significantly better at all follow-up points than did patients in the other two conditions – a substantial advantage of about 10 percentage points that endured across 3 years [5]. On this common measure, at least, the three treatments definitely were not equal in outcome. Furthermore, significant therapist effects on outcome were observed, even after controlling for patient and site characteristics [6]. It made a difference who delivered the treatment.

It is true that most of the primary patient-treatment matching predictions were not confirmed in early follow-up; instead, some large predicted matching effects were found with *a priori *hypothesis tests that had been assigned to secondary status [7], or emerged at 3-year follow-up [3]. Angry patients did better in Motivational Enhancement Therapy, those who lacked social support for abstinence fared best with Twelve Step Facilitation, and in all, five *a priori *hypotheses were supported [8]. To support their inaccurate claim that "essentially no patient-treatment matches were found," Cutler and Fishbain observed that "some 504 hypotheses were tested." The primary and secondary matching hypotheses numbered 19, however, all of which had been carefully specified *a priori*, with statistical protection against Type I error.

More generally, the MATCH study is widely recognized as setting a high standard for methodological rigor, treatment integrity, follow-up retention, and data verification, all of which should increase confidence in its findings. It is therefore unclear on what basis Cutler and Fishbain characterize the Project MATCH findings as "disappointing."

## Attacking a straw man

Their disappointment is tangential, however, to the principal aim of this article. Central to the authors' exposé tone is their claim that MATCH findings were interpreted as evidence that all three treatments were quite effective. The Project MATCH Research Group never asserted such efficacy claims, and explicitly cautioned against such interpretation [9]. The only evidence provided for the denounced post-hoc interpretation is a comment by then-Director of NIAAA, Dr. Enoch Gordis, quoted second-hand from *a Science News *article. From this single verbal remark, the authors implied widespread misrepresentation of the MATCH data. They quoted no MATCH scientific report as suggesting absolute efficacy of any of the treatments, because no such interpretation was made. Reaching farther, the authors misrepresented a published article by Miller, Bennett and Walters [2] as suggesting "that treatment is extremely effective by way of presenting the MATCH results." We made no such claim or suggestion in this article, and instead described the typical course of outcomes after treatment without drawing causal inference, offering this specific caveat (p. 219): "We caution that one cannot confidently assert from these averages that treatment caused the observed outcomes."

## Fundamental errors of logic

Their straw man notwithstanding, can the authors' own re-analyses of MATCH show that treatment was ineffective? Beyond the well-known logical problems with proving a null hypothesis, they attempted to do so based on a study that included no untreated control condition and thus cannot be used to judge the absolute efficacy of any of the treatments tested. The authors thereby ironically committed the very logical inference error for which they erroneously critiqued the MATCH investigators: that of interpreting correlational data to infer causality. If the MATCH study could not logically support any claim of absolute efficacy, how then could null findings within the same study prove a lack of efficacy?

Furthermore, the authors concluded that participants who reduce their alcohol consumption are more likely to enter or remain in treatment and those who continue drinking are more likely to drop out of treatment. To support this claim, they cited the Rand study [10] that reported (as typically occurs) a positive correlation between length of stay/retention and treatment outcome, which the Rand authors acknowledged could be due to selection bias: those who are more motivated will stay in treatment longer and also do better. However, this is precisely the opposite of what Cutler and Fishbain concluded from MATCH data – that length of stay was not substantially related to better outcomes. Indeed, their central argument is that patients did just as well when they only completed intake and no treatment. That is an exception to the usual finding, that retention is highly correlated with outcome. Thus they explained their principal finding, that length of treatment did not predict outcome, by arguing that length of treatment does predict outcome because of self-selection factors. In the process they again confused correlation with causality. If one cannot infer from corrrelations between attendance and outcome that a treatment worked, then surely one cannot infer from non-significant correlations (including their comparisons of patients who completed 0, 1, or all 12 sessions) that treatment was ineffective (thus proving a null hypothesis).

There is a further logical error in inferring that if behavior change was present at Week 1 and was maintained at Week 12, then treatment must have had no effect. One reason why behavior change appears so rapidly in alcohol treatment is that many patients initiate abstinence at the very beginning of treatment. Unlike the gradual reduction that one might expect in treating obesity or depression, the full behavior change appears abruptly with the onset of abstinence, and the principal challenge is to maintain that change. The authors seemed surprised by this "instantaneous" change, but in fact short-term abstinence is not all that difficult to achieve. The real challenge with all addictive behaviors is maintenance or "relapse prevention" [11]. Treatment ineffectiveness would be manifest in a short-term change that failed to be maintained over time. Maintaining initial gains is a hallmark of effective, not ineffective treatment.

There is also a problem in eliminating two-thirds of the MATCH sample before conducting the primary between-group comparison: that between patients completing 0, 1, or 12 sessions (the latter necessarily eliminating the 4-session MET condition). One explanation offered during review was the desire to keep it simple for readers, but how persuasive are analyses predicated on one-third of cases? If indeed their findings hold up when including the entire sample, why not do so?

## Exaggerated claims

The authors' misinterpretations are compounded in the discussion section, where their conclusions become still more expansive. For example: "Ineffective treatment would be the most parsimonious explanation for the rather surprising main findings of Project MATCH, that there was no match between patient characteristics and different types of treatment, and that all three treatments were equal." They asserted that their analyses show "that current treatments are not effective," and "that three of the best treatments currently available for addiction (sic) were not very effective." This again is flagrant use of correlational data to infer causality, and worse to prove the null hypothesis. This error is not mitigated by the use of qualifying verbs like "suggest" to justify a point that the authors wanted to make. The authors faulted Dr. Gordis for a single remark inferring causality from correlational data in MATCH, but predicated their own entire argument on the same flawed logic, claiming that low correlation demonstrates lack of efficacy.

Then out of the blue in the discussion section comes the claim "that part of the effect is not real; many active alcoholics underreport drinking." There is a large literature on the validity of self-report of alcoholics in outcome studies, rather consistently indicating that self-report yields reliable indices of drinking that are at least as high as those resulting from confirmatory measures. None of this literature was cited, and instead the authors simply implied that alcoholics lie. Furthermore, in order to produce the "unreal" result that the authors claimed, the participants in MATCH would have had to under-report their drinking significantly more at follow-up (after treatment) than at baseline. Project MATCH included objective measures (breath, blood tests) as well as collateral reports to confirm patient self-report – as extensive a check on self-report as done in any clinical trial of alcohol treatment. These measures all supported the validity of self-report. In defense of their claim that outcomes can be explained by patient under-reporting, the authors offered only the Rand finding "that 30% of the collateral informants were unable to provide information." This means, of course, that in 70% of cases collaterals did provide information, which by the way showed high agreement with patient self-report [10].

In their discussion, Cutler and Fishbain repeated the unsubstantiated claim that MATCH results were misrepresented as proving treatment efficacy. While it is hard to disagree with their pronouncement that "Exaggerated claims of treatment effectiveness can have undesirable consequences," what does this have to do with the MATCH study? It is equally true that exaggerated claims of treatment ineffectiveness can be harmful, and should be avoided.

## Implications

After repeated statements that the treatments in MATCH and treatments for alcoholism more generally are ineffective, the authors oddly allowed that "We are not suggesting that alcoholism treatment should be discontinued or even reduced." Would that not be a logical extension of their argument, particularly in an era of managed care? And what exactly, then, would be the "profound influence on alcoholism research and treatment" that the authors immodestly claimed should result from "widespread acceptance" of their re-interpretation of the Project MATCH data? Against the evidence of hundreds of randomized clinical trials demonstrating the efficacy of treatment methods for alcohol and other drug use disorders, Cutler and Fishbain offer only their belief that the absence of a large correlation between retention and outcome in the MATCH study proves the null hypothesis that "current psychosocial treatments" are ineffective. The above logical flaws and scientific misstatements were all pointed out during the review process, but the authors chose to retain their claims. Along the way, the press to support a negative conclusion far outstripped anything that reasonable scientists would conclude from these re-analyses.

## Competing interests

Dr. Miller was a Principal Investigator and the first Steering Committee Chair for the Project MATCH Research Group, funded by the National Institute on Alcohol Abuse and Alcoholism (NIAAA). He is also the senior author of one of the treatments tested in Project MATCH, described in *Motivational Enhancement Therapy *[12] and *Motivational Interviewing *[13], from the latter of which he derives authorship royalties.

## Pre-publication history

The pre-publication history for this paper can be accessed here:


